# Rod Photoreceptor Ribbon Synapses in DBA/2J Mice Show Progressive Age-Related Structural Changes

**DOI:** 10.1371/journal.pone.0044645

**Published:** 2012-09-05

**Authors:** Michaela Fuchs, Michael Scholz, Anna Sendelbeck, Jenny Atorf, Christine Schlegel, Ralf Enz, Johann Helmut Brandstätter

**Affiliations:** 1 Department of Biology, Animal Physiology, FAU Erlangen-Nuremberg, Erlangen, Germany; 2 Institut für Anatomie Lehrstuhl II, FAU Erlangen-Nuremberg, Erlangen, Germany; 3 Department of Ophthalmology, University Hospital Erlangen, Erlangen, Germany; 4 Institute for Biochemistry, FAU Erlangen-Nuremberg, Erlangen, Germany; Institut de la Vision, France

## Abstract

The DBA/2J mouse is a commonly used animal model in glaucoma research. The eyes of DBA/2J mice show severe age-related changes that finally lead to the degeneration of retinal ganglion cells and the optic nerve. Recent electroretinogram studies identified functional deficits, which suggest that also photoreceptor cells are involved in the pathological processes occurring in the DBA/2J mouse retina. In a comparative study, we examined anatomical and molecular changes in the retinae of DBA/2J and C57BL/6 control mice with light and electron microscopy and with PCR analyses. In the retina of the DBA/2J mouse, we found a thinning of the outer plexiform layer, the first synaptic layer in the transfer of visual signals, and age-dependent and progressive degenerative structural changes at rod photoreceptor ribbon synapses. The structural ribbon changes represent a photoreceptor synaptic phenotype that has not yet been described in this animal model of secondary angle-closure glaucoma. Furthermore, genes of the classical complement cascade were upregulated in the photoreceptor cells of aging DBA/2J mice, suggesting a putative link between ribbon synapse degradation and the innate immune system.

## Introduction

The DBA/2J mouse is a commonly used inbred strain for glaucoma research. The eyes of DBA/2J mice show severe age-related changes that include iris stroma atrophy and pigment dispersion, increase of intraocular pressure (IOP), and degenerative changes of retinal ganglion cells (RGC) and the optic nerve [1]–[3]. Because of these changes, the DBA/2J mouse serves as an accepted animal model of secondary angle-closure glaucoma [4], and an extensive literature exists, describing the degree and distribution of RGC degeneration and optic nerve damage in the DBA/2J mouse [2], [3], [5], [6].

The relevance of increased IOP as a diagnostic risk factor for glaucoma is beyond controversy, however, correlations of long-term IOP progression with axon loss in individual eyes show that in DBA/2J mice ocular hypertension cannot be considered the only causative factor for the onset of glaucomatous changes [3], [7]–[9]. Furthermore, recent studies examining the visual function in DBA/2J mice with electroretinogram (ERG) recordings showed functional deficits in the DBA/2J mouse retina, which were not correlated with changes in the IOP [10], [11]. The results of these studies suggest that in addition to the degeneration of RGCs, photoreceptor cells are affected by the pathological processes occurring in the retina of DBA/2J mice.

In this study, we report age-dependent and progressive structural changes at the ribbon synapses in the synaptic terminals of rod photoreceptor cells in the DBA/2J mouse retina. The ultrastructural changes were accompanied by a reduction in the thickness of the synaptic outer plexiform layer (OPL). Here, at the first synaptic layer of the retina, the photoreceptor cells transfer the visual signals to the post-receptoral retinal network, and malfunctioning ribbon synapses will lead to impaired vision or even total blindness. Finally, the results of our study show an age-dependent upregulation of the complement factor C1q in the photoreceptor cells of DBA/2J mice, suggesting a putative link between the ribbon synaptic phenotype and the innate immune system.

## Results

### The Width of the Synaptic OPL is Reduced in DBA/2J Mice

**Figure 1:**
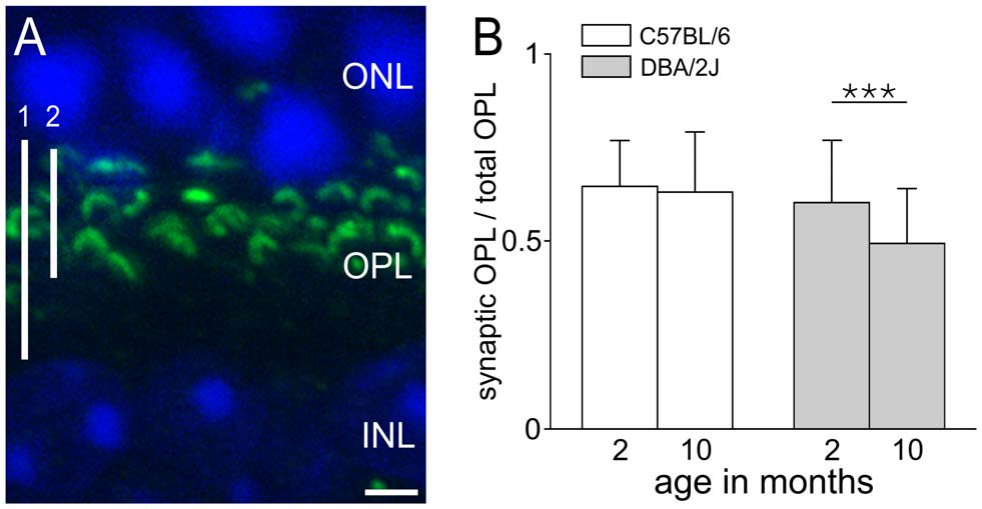
Reduced OPL width in aging DBA/2J mice. (A) The extension of the total outer plexiform layer (OPL; 1) and the synaptic OPL (2) was measured in vertical retinal sections stained with DAPI (blue) and an antibody against RIBEYE (green). (B) The synaptic OPL/total OPL ratio did not change in 2 to 10 months old C57BL/6 control mice, but significantly decreased in the age-matched DBA/2J mice (*** p<0.001). ONL, outer nuclear layer; INL, inner nuclear layer. Scale bar: 2 µm.

**Figure 2:**
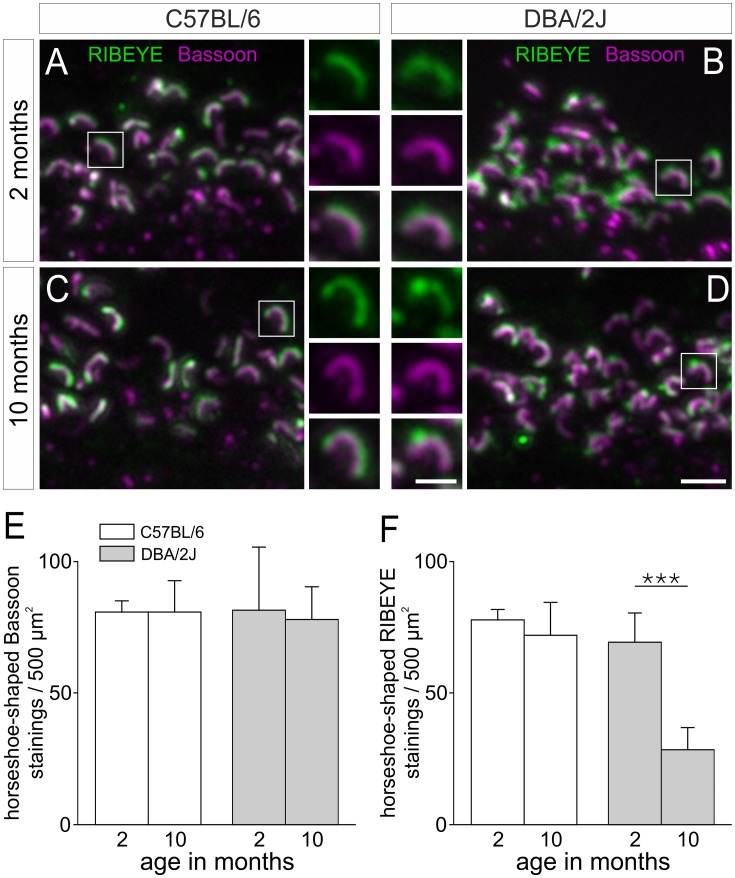
Altered photoreceptor synaptic ribbons in aging DBA/2J mice. (A–D) Representative examples of regions of the OPL of C57BL/6 (A,C) and DBA/2J retinae (B,D) double labeled for RIBEYE (green) and Bassoon (magenta). The boxes indicate the selected rod photoreceptor ribbons, which are shown at higher magnification. Whereas in the C57BL/6 retinae the rod photoreceptor ribbons displayed their typical horseshoe shape (A,C), the photoreceptor ribbons in the DBA/2J retinae lost their compact appearance as indicated by RIBEYE staining (B,D). This is most obvious in 10 months old DBA/2J mice (D). (E,F) Quantification of the number of horseshoe-shaped Bassoon (E) and RIBEYE (F) stainings in 500 µm^2^ OPL areas. Whereas the number of horseshoe-shaped Bassoon stainings did not change with age in DBA/2J and C57BL/6 control mice, the number of horseshoe-shaped RIBEYE stainings significantly decreased in aging DBA/2J mice (*** p<0.001). Scale bars: 2 µm (for A–D) and 1 µm (for inserts).

Because of the reported functional deficits in the outer retina of the DBA/2J mouse [10], [11], we searched for possible morphological alterations at the level of the OPL, the first synaptic layer in the transfer of visual signals from the photoreceptor cells to their postsynaptic bipolar and horizontal cells. In a comparative analysis, we examined vertical sections of 2 to 10 months old DBA/2J and C57BL/6 control retinae doubly stained with DAPI (blue) to label the cell somata and with an antibody against RIBEYE (green) to label the photoreceptor synaptic ribbons (Fig. 1). First, we measured the width of the total OPL, which corresponds to the distance from the innermost row of the nuclei of the photoreceptor cells to the outermost row of the nuclei of the bipolar and horizontal cells, and includes the photoreceptor terminals and the postsynaptic plexus of the bipolar and horizontal cell dendrites and processes (Fig. 1A; 1). Next, we assessed the width of the synaptic OPL, which corresponds to the area occupied by the RIBEYE-stained photoreceptor ribbons (Fig. 1A; 2). Finally, the ratios of the synaptic OPL divided by the total OPL were calculated and compared between 2 and 10 months old DBA/2J and C57BL/6 control mice (Fig. 1B). No changes were found in the width of the synaptic OPL with increasing age in the C57BL/6 control mice (Fig. 1B). In the DBA/2J mice, however, the width of the synaptic OPL significantly decreased from 2 to 10 months of age (Fig. 1B; p<0.001). As the thickness of the outer (ONL) and inner nuclear layer (INL) did not differ between ages and between the two mouse strains (data not shown), the reduction of the synaptic OPL in the DBA/2J retina is most likely not caused by a loss of photoreceptor cells or their postsynaptic bipolar and horizontal cells.

### Rod Photoreceptor Ribbon Structure is Altered in DBA/2J Mice

**Figure 3:**
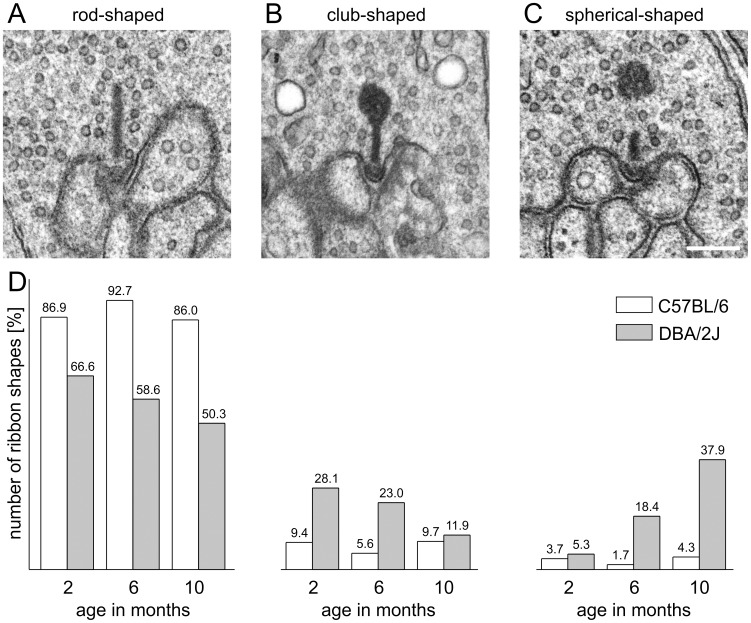
Rod photoreceptor synaptic ribbons disintegrate in aging DBA/2J mice. Synaptic ribbon profiles were classified as rod-shaped (A), club-shaped (B), and spherical-shaped (C). (D) Number of ribbon shapes in retinae of 2, 6, and 10 months old DBA/2J and C57BL/6 control mice. In the C57BL/6 control retinae the majority of the rod photoreceptor synaptic ribbon profiles was rod-shaped at all ages. In the DBA/2J retinae the number of rod-shaped ribbon profiles decreased and the number of spherical-shaped ribbon profiles increased with age. Scale bar: 0.2 µm (for A–C).

Next, we examined in detail with high resolution light and electron microscopy the photoreceptor ribbon synapses in the OPL of DBA/2J and C57BL/6 control mice (Figs. 2 and 3). The presynaptic ribbon complex of photoreceptor cells comprises at least two functionally distinct compartments: the electron dense synaptic ribbon and the arciform density/plasma membrane compartment [12], [13]. For the light microscopical analysis, we labeled two major proteins of the presynaptic ribbon complex: RIBEYE, the main structural component of the synaptic ribbon [14], and Bassoon, which is part of the arciform density and anchors the synaptic ribbon to the plasma membrane [12], [15]. In 2 to 10 months old C57BL/6 mice, most rod photoreceptor ribbons showed the well-known horseshoe-shaped staining for RIBEYE (green), which bends around the Bassoon staining (magenta) as seen in high magnification views of single ribbon synaptic sites (Fig. 2A and 2C). In the DBA/2J mice, Bassoon staining remained unchanged and comparable to the C57BL/6 retina, but the RIBEYE staining showed clear signs of disintegration (Fig. 2B and 2D). Most obvious, in older DBA/2J mice (10 months), but already observable in 2 months old mice, many synaptic ribbons showed structural alterations (Fig. 2B,D), and differed in their appearance from the synaptic ribbons in age-matched C57BL/6 mice (Fig. 2A and 2C).

In addition to the structural analysis, we quantified rod photoreceptor ribbon synaptic sites by counting horseshoe-shaped stainings for both Bassoon (marks the arciform density) and RIBEYE (marks the ribbon) in 500 µm^2^ areas of the OPL of aging DBA/2J and C57BL/6 control mice (Fig. 2E and 2F). The number of horseshoe-shaped Bassoon stainings did not differ in both mouse strains between 2 and 10 months (Fig. 2E), but the number of horseshoe-shaped RIBEYE stainings significantly decreased in the DBA/2J retina with increasing age (Fig. 2F; p<0.001). These data indicate that synaptic sites are not completely lost in the retina of aging DBA/2J mice, while the ribbons in rod photoreceptor terminals selectively undergo structural alterations.

Therefore, we investigated the morphology of synaptic ribbons in greater detail with electron microscopy. We photographed several thousand photoreceptor terminals and classified the synaptic ribbons according to their morphological appearance into rod-shaped, club-shaped, and spherical-shaped. Representative examples of the various ribbon shapes are shown in Figure 3A–C, and the quantitative data are summarized in Figure 3D. In 2 months old C57BL/6 mice, 86.9% of 1,069 rod photoreceptor ribbon profiles were presynaptically anchored and rod-shaped; the remaining ribbon profiles were club-shaped and anchored (9.4%) or spherical-shaped and free-floating (3.7%; Fig. 3D). In 6 and 10 months old C57BL/6 mice, the number of rod-shaped ribbon profiles was similar (6 months: 92.7% of 644 rod photoreceptor ribbons; 10 months: 86% of 671 rod photoreceptor ribbons), indicating no age-related changes in ribbon shape in the C57BL/6 mouse strain (Fig. 3D). In the DBA/2J mice, on the other hand, only 66.6% of 1,108 rod photoreceptor ribbon profiles were presynaptically anchored and rod-shaped at the age of 2 months, while a substantial number of ribbon profiles was club-shaped (28.1%); the number of spherical-shaped profiles (5.3%) in 2 months old DBA/2J mice was comparable to the number found in the C57BL/6 mice (Fig. 3D). From 6 to 10 months, the number of rod-shaped ribbon profiles decreased in the DBA/2J mice (6 months: 58.6% of 860 rod photoreceptor ribbons; 10 months: 50.3% of 700 rod photoreceptor ribbons), as did the number of club-shaped ribbon profiles (6 months: 23%; 10 months: 11.9%). In contrast, the number of spherical-shaped ribbon profiles increased with age (6 months: 18.4% of 860 rod photoreceptor ribbons; 10 months: 37.9% of 700 rod photoreceptor ribbons). The 7-fold increase in the number of free-floating, spherical-shaped ribbon profiles from 2 to 10 months, and the concomitant decrease in the number of anchored, rod-shaped ribbon profiles, clearly indicates a degradation of rod photoreceptor ribbons with progressing age in the DBA/2J mouse (Fig. 3).

Empty terminals, i.e. terminals in single ultrathin sections without ribbon structures, were also counted. Their numbers did not differ between strains and ages (data not shown), corroborating the finding that synaptic sites were not completely lost in the DBA/2J retina (Fig. 2E). Furthermore, in cone photoreceptor terminals of both mouse strains and at all examined ages, the prominent ribbon form was rod-shaped and only very few club-shaped and no spherical-shaped ribbon profiles were found (data not shown).

### Retinal Structure and Neuronal Morphologies are Comparable between DBA/2J and C57BL/6 Control Mice

**Figure 4:**
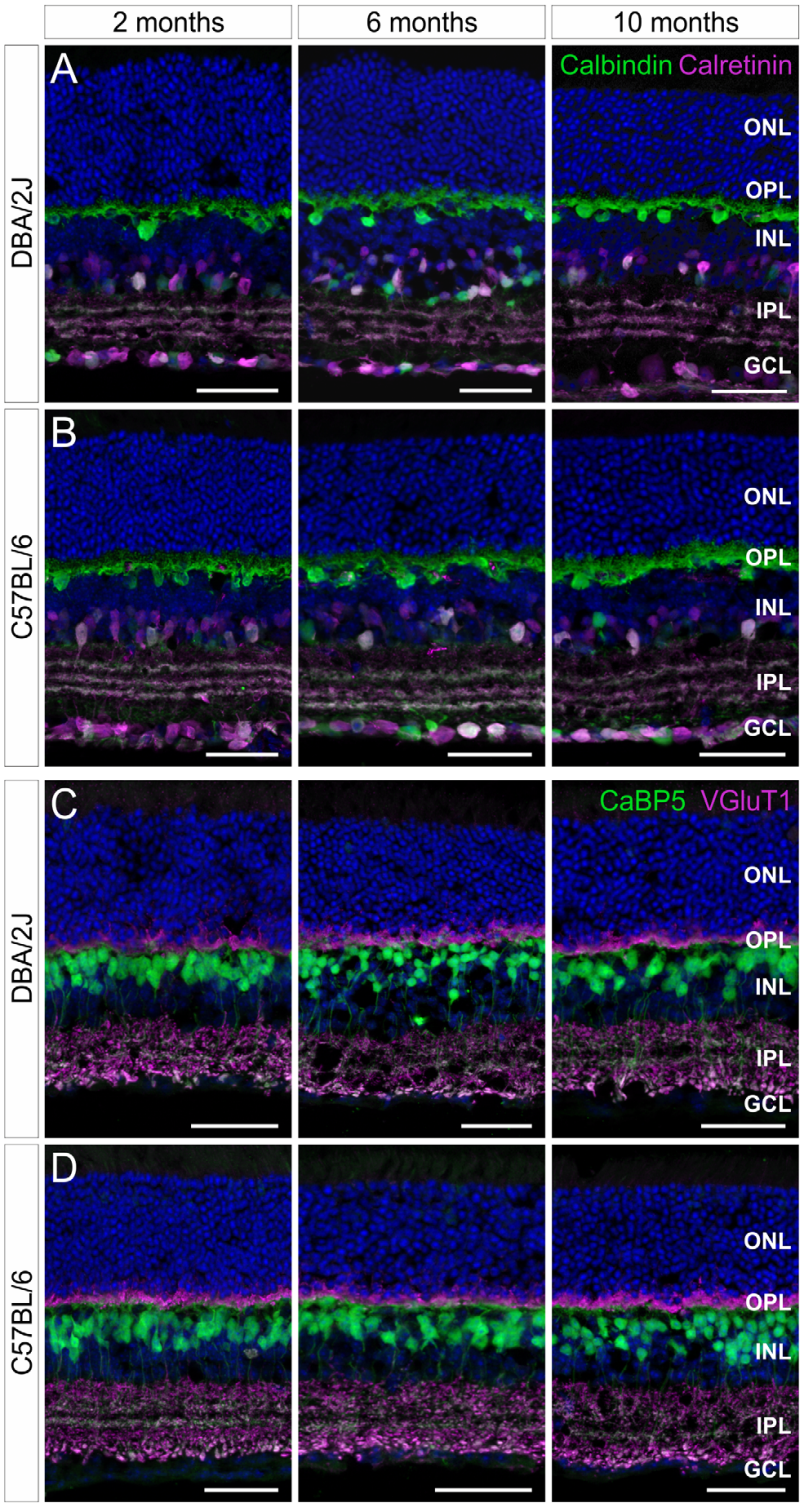
No changes in retinal structure and neuronal morphologies in aging DBA/2J mice. Double labelings with antibodies against Calbindin (green) and Calretinin (magenta) (A,B) or against CaBP5 (green) and VGluT1 (magenta) (C,D) show no obvious differences in retinal structure and neuronal morphologies between aging DBA/2J and C57BL/6 control mice. Retinal layers are indicated (OS, outer segments; IS, inner segments; ONL, outer nuclear layer; OPL, outer plexiform layer; INL, inner nuclear layer; IPL, inner plexiform layer; GCL, ganglion cell layer). Scale bar: 20 µm.

In a further analysis, we performed a series of immunocytochemical experiments to look for possible alterations in retinal structure and neuronal morphologies in the aging DBA/2J mouse retina. Vertical cryostat sections of DBA/2J and C57BL/6 control retinae at 2, 6, and 10 months of age were triple labeled with antibodies against various types of retinal neurons and with DAPI to label the cell somata (Fig. 4). Horizontal cells, amacrine cells, displaced amacrine cells and ganglion cells with their processes stratifying in the OPL and the inner plexiform layer (IPL), respectively, were stained with antibodies against the calcium-binding proteins Calbindin (green) and Calretinin (magenta; Fig. 4A and 4B). Rod and cone bipolar cells with their dendrites contacting rod and cone photoreceptor cells in the OPL and with their axons terminating in the OFF and ON sublamina in the IPL were stained with an antibody against the calcium-binding protein CaBP5 (green; Fig. 4C and 4D). Finally, an antibody against the vesicular glutamate transporter 1 (VGluT1) was used to label the glutamatergic synaptic terminals of rod and cone photoreceptor cells in the OPL, and of OFF and ON bipolar cells in the IPL (magenta; Fig. 4C and 4D). As depicted in Figure 4, retinal structure and the morphology of the various retinal neurons and their stratification patterns in the two synaptic layers of the retina, the OPL and IPL, were similar at all examined ages and did not differ between DBA/2J and C57BL/6 control mice (Fig. 4).

### Genes of the Classical Complement Cascade are Upregulated in Photoreceptor Cells of DBA/2J Mice

It was previously shown that C1qA and C1qB, proteins of the classical complement cascade, are upregulated and synaptically relocalized in the adult DBA/2J mouse retina [16], [17]. Especially the findings of C1q mRNA in RGCs and of C1q proteins at synapses in the IPL, as well as the increasing C1q mRNA levels with increasing age in the DBA/2J retina, suggested that complement-mediated synapse elimination may be involved in RGC degeneration in glaucoma [17].

**Figure 5:**
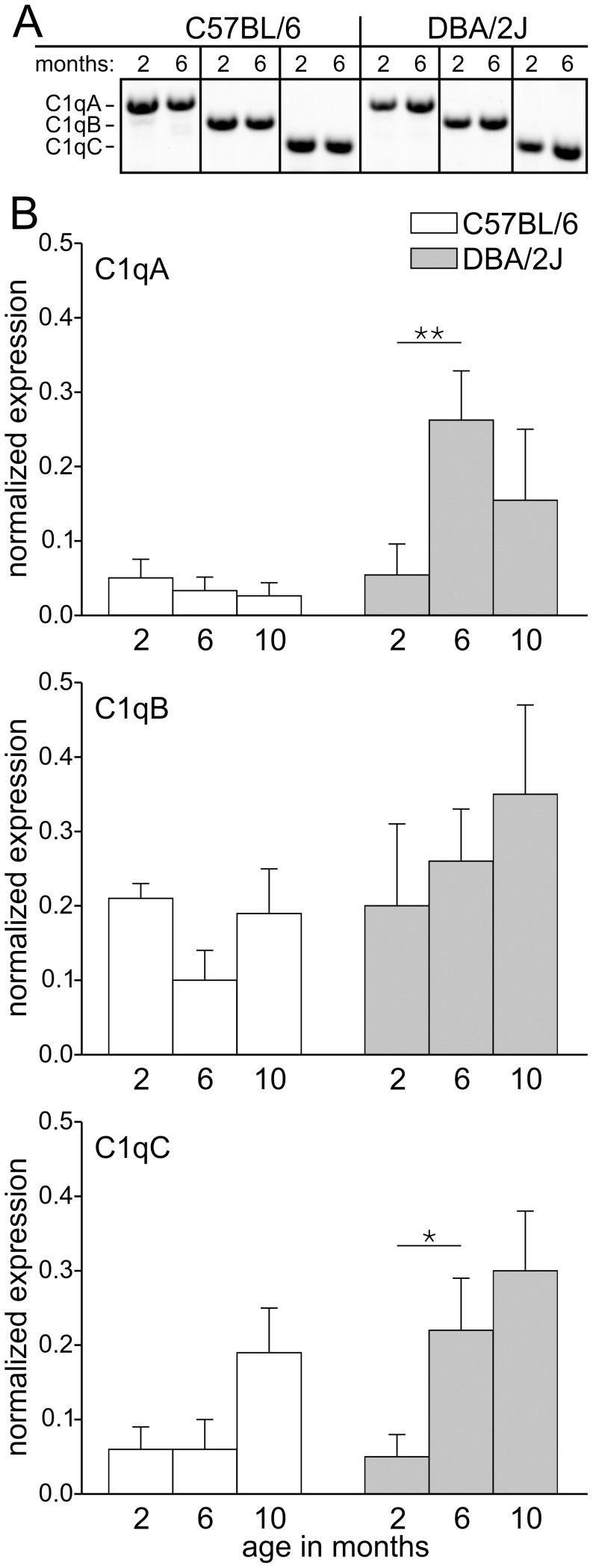
C1qA and C1qC gene expression is upregulated in photoreceptor cells of aging DBA/2J mice. (A) Agarose gels showing PCR fragments that represent the gene expression of C1qA, C1qB and C1qC in the retinae of 2 and 6 months old DBA/2J and C57BL/6 control mice. (B) The expression of C1qA, C1qB and C1qC in photoreceptor cells of 2, 6, and 10 months old DBA/2J mice and age-matched C57BL/6 control mice was compared with PCR. For each age, cDNA obtained from three animals was subjected to triplicate PCR amplifications (n = 9). Statistically significant differences are indicated by asterisks (* p<0.05; ** p<0.01).

We therefore asked whether C1q proteins are present also in the photoreceptor cells of the adult DBA/2J mouse retina, and whether C1q protein levels increase with age. Unfortunately, available antibodies against C1q proteins did not work in our hands. Therefore, we decided to examine C1q gene expression in aging DBA/2J and C57BL/6 control mice. First, we ensured that we could reliably detect C1qA, C1qB, and C1qC transcripts in the adult DBA/2J and C57BL/6 retina by RT-PCR (Fig. 5A). To analyze the gene expression specifically in photoreceptors, we then collected pieces of the ONL containing the photoreceptor somata by laser capture microdissection from retinal cryostat sections of 2, 6, and 10 months old DBA/2J and C57BL/6 control mice. DNA fragments representing C1q transcripts were generated in triplicate PCR experiments using cDNA preparations from three different retinae for each age and mouse strain. Comparing the expression of C1q transcripts in the ONL of 2, 6, and 10 months old mice demonstrated a significant upregulation of C1qA (p = 0.009) and C1qC (p = 0.027) in DBA/2J mice from 2 to 6 months (Fig. 5B). A similar tendency was found for C1qB expression (Fig. 5B), but not for C57BL/6 control mice (Fig. 5B). Calculated ratios of the obtained values for C1qA, C1qB, and C1qC are shown in Table 1.

**Table 1:** Changes of C1q transcript levels.

x-fold change (2 → 6 months)	C57BL/6	DBA/2J
C1qA	−1.67±1.07	5.20±1.07
C1qB	−2.10±0.50	1.30±0.82
C1qC	1.00±1.17	4.40±0.92

Calculated ratios of C1q gene expression between 2 and 6 months old animals. A 2-fold increase in mRNA concentration indicates a 100% upregulation of the corresponding gene. The upregulation of C1qA and C1qC in the ONL of 6 months old DBA/2J mice is clearly visible.

## Discussion

The DBA/2J mouse is a widely used animal model of ocular hypertension, and it is well established that aging DBA/2J mice exhibit RGC degeneration and optic nerve damage [1]–[3]. In this study we present evidence for the degradation of presynaptic ribbons in rod photoreceptor terminals of the DBA/2J mouse, a phenotype that has not yet been described in this animal model of glaucoma.

Recent reports about functional deficits in the DBA/2J retina involving photoreceptor cells [10], [11] motivated us to examine the photoreceptor terminals and their ribbon synapses in the OPL of the DBA/2J retina in detail. We found a reduction in the extension of the synaptic OPL in aging DBA/2J mice, and structural alterations and a loss of RIBEYE-stained horseshoe-shaped rod photoreceptor ribbons. However, there was no complete loss of synaptic sites in the DBA/2J retina, as seen in the persisting horseshoe-shaped Bassoon staining, which marks the arciform density. This behavior of the two proteins RIBEYE and Bassoon is well in accordance with results from a recent study of our group describing non-stable (RIBEYE) and stable (Bassoon) components of the photoreceptor ribbon complex [18]. The subsequent ultrastructural analysis verified the light microscopical findings of ribbon structural changes with the occurrence of club-shaped and spherical-shaped free-floating ribbon profiles in the DBA/2J retina. These structural changes became more obvious with increasing age. Although we have no explanation for it, it is noteworthy that in the DBA/2J retina ribbon disassembly was only observed in rod but not cone photoreceptor terminals.

Free-floating, spherical-shaped ribbon profiles have been described as ribbon precursors during photoreceptor synaptogenesis [19], [20], and also as products of ribbon degradation and a sign of pathological processes occurring in photoreceptor cells and at their synapses. For example, in mouse photoreceptor terminals deficient of the cytomatrix protein Bassoon, synaptic ribbons are not anchored to the active zone and disintegrate into spherical-shaped ribbon material [15], [21]. A similar ribbon synaptic phenotype is found in mouse photoreceptor terminals deficient of the Ca^2+^-binding protein CaBP4, which modulates the activity of L-type Ca_v_1.4 channels present at rod photoreceptor active zones [22], the protein Tulp1, which is involved in intra-photoreceptor ciliary transport processes [23], or the SNARE regulatory proteins Complexin 3 and 4 [24].

Structural alterations of presynaptic ribbons impair synaptic transmission, and the ribbon changes found in rod photoreceptor terminals of the DBA/2J retina correlate well with results of recent ERG studies showing that aging DBA/2J mice exhibit reduced amplitudes of the scotopic a- and b-wave [10], [11]. The a-wave mainly reflects the photocurrent of rod and cone photoreceptor cells in response to changing light conditions, while the b-wave reflects mainly ON bipolar cell activity and thus is an indicator of ribbon synaptic function [25].

Expression analyses in the glaucomatous retina of DBA/2J mice identified several proteins putatively involved in the apoptotic cell death of RGCs, including receptors for glutamate and ryanodine, epithelial sodium channels and endothelin 2 [26]–[29]. From the results of our study it becomes clear that in addition to the known glaucomatous changes in RGCs leading to optic nerve degeneration also photoreceptor ribbon synaptic structure in the retina of aging DBA/2J mice is affected. But what might be the cause for the rod photoreceptor ribbon synaptic phenotype in the DBA/2J retina? Putative candidates are components of the complement cascade, which play an important role in the controlled removal of apoptotic cells and are also implicated in the elimination of supernumerary synapses in the developing visual system [30]. Recent studies showed that the protein C1qB, which is the initiating protein in the classical complement cascade and part of the innate immune system, was upregulated in the retina of the DBA/2J mouse [16]. Another study reports an upregulation of C1qA and C1qB in RGCs even before or concurrent with RGC degeneration, accompanied by the appearance of C1q proteins at the postsynaptic sites of RGC dendrites in the IPL of the glaucomatous retina [17]. Furthermore, microarray studies comparing gene expression profiles of healthy and glaucomatous retinae of DBA/2J mice identified components of the complement cascade [31], [32].

With increasing age, DBA/2J mice show severe iris atrophy [33] leading to an unphysiological high retinal illuminance and metabolic stress, which in turn might cause a degradation of rod photoreceptor synaptic ribbons. In our study, we found increased gene expression for C1qA and C1qC in the photoreceptor cells of aging DBA/2J mice suggesting an involvement of the classical complement system in ribbon degradation. However, it remains to be shown whether there is a direct link between C1q upregulation in photoreceptor cells and the degradation of rod photoreceptor synaptic ribbons in the DBA/2J mouse.

### Concluding Remarks

Here we present a so far undescribed structural photoreceptor ribbon phenotype in the retina of the DBA/2J mouse, an accepted and commonly used animal model in glaucoma research. As there is no reported photoreceptor involvement in the pathology of human glaucoma, this finding may question the suitability of the DBA/2J mouse in glaucoma research. However, irrespective of its use in glaucoma research, the DBA/2J mouse serves as a good animal model for the study of retinal and thus central nervous system related degenerative processes.

## Materials and Methods

### Ethics Statement

The experiments were performed in compliance with the guidelines for the welfare of experimental animals issued by the Federal Government of Germany and the FAU Erlangen-Nuremberg. The animal experiments were approved and registered by the Amt für Veterinärwesen der Stadt Erlangen (AZ: TS - 10/07 Lehrstuhl für Zoologie-Tierphysiologie). Mouse breeding was performed in the animal facilities of the FAU University of Erlangen-Nuremberg according to European and German (Tierschutzgesetz) guidelines for the welfare of experimental animals (AZ 820-8791.2.63).

### Animals

For this study, DBA/2J and C57BL/6 mice at 2, 6, and 10 months of age were kept on a 12/12 hour light/dark cycle with light on at 06∶00 am (average light intensity 200 lux) and with food and water ad libitum.

Retinal tissue preparation for light microscopic immunocytochemistry and quantitative analysis of the OPL width and of the number of ribbons and synaptic sites.

Mice were deeply anesthetized with isoflurane and killed by cervical dislocation. The eyes were enucleated and the cornea and lens were rapidly removed. The eyecups were fixed for 30 minutes in 4% paraformaldehyde in phosphate buffer (PB, 0.1 M, pH 7.4). Fixed retinae were removed from the eyecup and cryoprotected in increasing concentrations of sucrose in PB (10%, 20%, 30%) at 4°C before being frozen in freezing medium (Tissue-Tek, Sakura, Netherlands). Vertical sections (12 µm) were cut on a cryostat (Leica Microsystems, Nussloch, Germany), collected on slides, and stored at −20°C.

Immunocytochemical labeling was performed by the indirect fluorescence method. The retinal sections were blocked for 1 hour in blocking solution (10% normal goat serum, 0.5% Triton X-100 in PB) and incubated in the primary antibodies (diluted in 3% normal goat serum, 0.5% Triton X-100 in PB) overnight at room temperature. After 3 times washing in PB, sections were incubated with secondary antibodies coupled to AlexaFluor 488 or AlexaFluor 594 (1∶500; Invitrogen, Darmstadt, Germany) and with DAPI (4,6-diamidino-2-phenylindole; 1∶20,000; Sigma-Aldrich, St. Louis, MO) for 1 hour at room temperature in the dark. Images were taken with a Zeiss Axio Imager.Z1 equipped with an ApoTome (Zeiss, Oberkochen, Germany). Images were adjusted for contrast and brightness using Adobe Photoshop CS5 (Adobe Systems, Munich, Germany).

The following antibodies were used: monoclonal mouse anti-Bassoon (1∶2,500; Stressgen, Victoria, Canada; [15], [20]), mouse anti-Calretinin (1∶2,000; Millipore, Schwalbach, Germany; [34]), polyclonal rabbit anti-CaBP5 (1∶1,000; [24], [35]), rabbit anti-Calbindin (1∶1,000; Swant, Marly, Switzerland; [36]), rabbit anti-RIBEYE (1∶1,000; Synaptic Systems, Göttingen, Germany; [18]), and polyclonal guinea pig anti-VGluT1 (1∶50,000; Millipore, Schwalbach, Germany; [20]).

The width of the total OPL (distance from the innermost row of the photoreceptor nuclei to the outermost row of the bipolar and horizontal cell nuclei) and of the synaptic OPL (represented by the RIBEYE-stained photoreceptor ribbons) was measured with ImageJ (Rasband WS, 1997–2012, National Institutes of Health, Bethesda, Maryland, USA; http://imagejnihgov/ij/). From vertical sections of 3–4 different retinae, 10 images across the entire retina were taken for each age and mouse strain. Measurements of the total and synaptic OPL were taken at intervals of at least 10 µm; C57BL/6: 2 months (n = 79), 10 months (n = 95), DBA/2J: 2 months (n = 94), 10 months (n = 97). To quantify the number of ribbons and synaptic sites in the OPL of 2 and 10 months old DBA/2J and C57BL/6 control mice, horseshoe-shaped RIBEYE and Bassoon stainings were counted in 500 µm^2^ areas (n = 6 for each age). Data are expressed as mean ± SD. Statistical significances were evaluated with the Kruskal-Wallis one-way analysis of variance. Differences were considered significant at p<0.05.

### Retinal Tissue Preparation for Conventional Electron Microscopy and Quantification of the Electron Microscopical Data

For conventional electron microscopy, fixation of retinae was in 4% paraformaldehyde and 2.5% glutaraldehyde for 2 hours at room temperature. The contrasting was carried out by incubation in 1.5% potassium ferrocyanide and 2% osmium tetroxide in cacodylate buffer for 1.5 hours. Retinae were dehydrated by an ethanol series and propylene oxide with 0.5% uranyl acetate added at the 70% ethanol step. The tissue was embedded in Epon resin (Fluka, Buchs, Switzerland). Ultrathin sections (60 nm) were stained with uranyl acetate and lead citrate. The sections were examined and photographed using a Zeiss EM10 electron microscope (Zeiss) and a Gatan SC1000 OriusTM CCD camera (GATAN, Munich, Germany) in combination with the DigitalMicrograph™ software (GATAN, Pleasanton, CA). Images were adjusted for contrast and brightness using Adobe Photoshop CS5 (Adobe Systems). For each experimental condition we examined random ultrathin sections of retinae from 3 animals. For the quantification of synaptic ribbon states, we took images of the OPL at a magnification of × 20,000. Between 1,500 and 2,500 photoreceptor terminals for each age were examined and subsequently classified into several categories: (1) terminals with rod-shaped ribbon profiles, (2) terminals with club-shaped ribbon profiles, (3) terminals with spherical-shaped ribbon profiles, and (4) terminals without ribbons.

### Laser Capture Microdissection and RNA Isolation

For laser capture microdissection, whole eyes were embedded in freezing medium, rapidly frozen in liquid nitrogen cooled isopentane and stored at −80°C. Frozen sections (30 µm) were cut and collected on slides (MembraneSlide 1.0 PEN, Zeiss, Munich, Germany). Frozen sections were dehydrated in ice-cold 70% ethanol for 2 minutes to reduce RNase activity. Slides were washed in RNase-free water to remove excess freezing medium and stained for 20 seconds in ice-cold cresyl violet solution (1% in 50% ethanol). Excess stain was removed on an absorbent surface and with subsequent dipping in ice-cold 70% ethanol. Slides were air-dried at room temperature. Laser capture microdissection was performed with a PALM micro Beam system (Zeiss) equipped with a nitrogen laser (337 nm). Outer nuclear layer and inner segments of photoreceptor cells were cut and catapulted into caps (AdhesiveCap 500 µl, Zeiss). Total RNA was extracted from the captured tissue using the RNeasy Micro Kit (Qiagen, Hilden, Germany) and subjected to reverse transcription.

### CDNA Synthesis and PCR Amplification

Total RNA obtained from the laser microdissected ONL was preincubated with 200 ng random hexamers for 10 min at 70°C. The mRNA was then reverse transcribed in a total volume of 25 µl containing 200 Units M-MLV reverse transcriptase (Promega, Madison, WI), 24 U RNAsin, 1.25 µl 10 mM dNTPs, and 5 µl M-MLV RT 5x buffer. The samples were incubated for 10 min at room temperature followed by 1 h at 42°C. PCR amplification was performed using 1 µl cDNA as a template in 10 µl PCR buffer (20 mM Tris–HCl pH 8.0, 50 mM KCl, 2.5 mM MgCl_2_, 0.2 mM dNTPs, 2.5 mM, 1 U Taq-polymerase; Invitrogen) in a programmable thermocycler (GeneAmp PCR System 9700; Applied Biosystems, Foster City, CA) using primer pairs specific for C1qA (sense: 5′-agctgctggcatccggac-3′, antisense: 5′-ggtcccacttggagatcac-3′), C1qB (sense: 5′-cctgaggaccatcaacagc-3′, antisense: 5′-ctcctcttgctctagcttc-3′), and C1qC (sense: 5′-cgatacaaacagaagcaccag-3′, antisense: 5′-ctggcaaggttgaggttcag-3′) with the following parameters: 94°C for 2 minutes followed by 40 cycles at 94°C for 45 s, 62°C for 60 s, 72°C for 30 s and a final incubation at 72°C for 10 minutes. To compare cDNA concentrations between samples, primers for the house keeping gene EF1α (sense: 5′-gtctgcccagaaagctcag-3′, antisense: 5′-aatggtctcaaaattctgtgac-3′) were used under the same conditions. The identity of the PCR products was verified by DNA sequencing.

Five µl of each PCR product were analyzed on 1.5% agarose gels stained with GelRed (Biotium, Hayward, CA) and photographed using a computer assisted gel documentation system (ChemiDoc XRS+; Bio-Rad, Munich, Germany). Intensities of PCR signals were determined with ImageLab 2.0.1 (Bio-Rad) and further data analysis was performed with Origin 5.5 (Microcal Software Inc., Northampton, MA). To balance differences in the concentrations of cDNA preparations, all intensities were normalized to the corresponding values of EF1α. Each PCR was performed in triplicates and data are expressed as mean ± SEM. Statistical significances were evaluated with the Kruskal-Wallis one-way analysis of variance. Differences were considered significant at p<0.05. To ensure that detected intensity differences between PCR products represent differences in C1q expression, we tested (i) for the presence of contaminating chromosomal DNA in our cDNA samples and (ii) for carry-over contaminations between PCR amplifications. To exclude that our cDNA samples contained chromosomal DNA, an additional PCR was designed that would detect an intronic sequence of EF1α. Using a chromosomal DNA preparation of DBA/2J mice as a positive control, a sense primer annealing to the EF1α intron 7–8 (5′-taagcctgatctgagtctgc-3′) in combination with the above described EF1α antisense primer generated a DNA fragment of the predicted size, applying the above described temperature profile for 40 cycles. In contrast, none of our cDNA samples showed any detectable PCR product under the same experimental conditions, verifying that our RNA preparations were devoid of any chromosomal DNA contamination. To test for carry-over contaminations between PCR amplifications, controls without adding template were included that showed no detectable PCR products.

To amplify C1qA-C transcripts from complete DBA/2J and C57BL/6 mouse retinae, cDNA samples were generated using a previously described protocol [28]. Subsequently, 250 ng reverse transcribed RNA was used for PCR amplification in 20 µl PCR buffer applying the same temperature profile as described before for 35 cycles (C1qA-C).
